# Vitamin K-Dependent Proteins in Skeletal Development and Disease

**DOI:** 10.3390/ijms22179328

**Published:** 2021-08-28

**Authors:** Michael Stock, Georg Schett

**Affiliations:** 1 Department of Internal Medicine 3, Friedrich Alexander University Erlangen-Nürnberg and Universitätsklinium Erlangen, 91054 Erlangen, Germany; Georg.schett@uk-erlangen.de; 2 Zentrum für Immuntherapie, Friedrich Alexander University Erlangen-Nürnberg and Universitätsklinikum Erlangen, 91054 Erlangen, Germany

**Keywords:** vitamin K, vitamin K-dependent proteins, osteocalcin, matrix Gla protein, Ucma, GRP

## Abstract

Vitamin K and Vitamin K-dependent proteins (VKDPs) are best known for their pivotal role in blood coagulation. Of the 14 VKPDs identified in humans to date, 6 play also important roles in skeletal biology and disease. Thus, osteocalcin, also termed bone Gla-protein, is the most abundant non-collagenous protein in bone. Matrix Gla protein and Ucma/GRP on the other hand are highly abundant in cartilage. Furthermore, periostin, protein S, and growth arrest specific 6 protein (GAS 6) are expressed in skeletal tissues. The roles for these VKDPs are diverse but include the control of calcification and turnover of bone and cartilage. Vitamin K plays an important role in osteoporosis and serum osteocalcin levels are recognized as a promising marker for osteoporosis. On the other hand, matrix Gla protein and Ucma/GRP are associated with osteoarthritis. This review focuses on the roles of these three VKDPs, osteocalcin, matrix Gla protein and Ucma/GRP, in skeletal development and disease but will also summarize the roles the other skeletal VKDPs (periostin, protein S and GAS6) in skeletal biology.

## 1. Introduction

Vitamin K belongs to the group of fat-soluble vitamins. In 1929 it was discovered by the Danish biochemist Henrik Dam as a dietary component essential for blood coagulation [1]. This vitamin is further sub-grouped into the naturally occurring vitamins K1 and K2 [2]. Although still best known for its pivotal role in blood coagulation, Vitamin K has been identified to be involved in a variety of further physiological functions in the last decades.

Thus, vitamin K has been discovered to play crucial roles in cell growth and proliferation, apoptosis, oxidative stress, inflammatory processes, and calcification processes [3,4,5,6]. Dietary shortage of vitamin K has been associated with increased risk of cancer and cardiovascular disease [5,7,8]. Surprisingly, however, a recent study suggests that elevated uptake of vitamin K2 may be linked to increased risk for breast cancer and higher mortality in breast cancer patients [9].

In the mid-1970s severe cases of bone malformations have been found in newborns of women who had received anticoagulant therapy with the vitamin K antagonist warfarin during their pregnancy [10]. These cases of chondrodysplasia punctata or fetal warfarin syndrome were the first indications that vitamin K is also associated with skeletal development and biology. Fetal warfarin syndrome, which is characterized by excessive growth plate calcification, could be recapitulated in experimental animal models applying a high dose warfarin diet [11]. Later it became clear that these ectopic calcifications and subsequent skeletal malformations were predominantly a consequence of inadequate production of two vitamin K-dependent proteins: osteocalcin (also termed bone Gla-protein) and matrix Gla-protein (MGP). Osteocalcin and MGP are both highly expressed in skeletal tissues. Thus, osteocalcin is specifically expressed by osteoblasts, while MGP exhibits a somewhat broader expression pattern, with high expression in chondrocytes, but also in vascular smooth muscle cells and epithelial cells [12,13,14]. The biological significance of these two proteins has been unraveled by the investigation of gene-targeted knock-out mice. Thus, osteocalcin has been identified as a negative regulator of bone formation, while MGP has been shown to be an inhibitor of tissue calcification [11].

The biological properties of these skeletal proteins are dependent on a vitamin K-dependent post-translational modification: specific glutamic acid (Glu) residues are post-translationally converted into gamma-carboxyglutamic acid (Gla) residues via a vitamin K-dependent mechanism [13,15]. As a vitamin K antagonist warfarin inhibits gamma carboxylation of Glu residues of these proteins and thereby impairs their physiological function [2,16].

Since vitamin K has been uncovered to play a pivotal role in skeletal biology after unraveling the mechanism underlying the fetal warfarin syndrome further implications of vitamin K in skeletal biology have been uncovered. Thus, the effects of vitamin K independent of glutamyl γ-carboxylation have been identified. For example, vitamin K has been shown to modify gene expression by binding to the nuclear receptor steroid and xenobiotic receptor (SXR)/pregnane X receptor (PXR) and thereby modulate bone homeostasis [17,18]. Gla-independent suppression of NFκB signaling in osteoblasts and osteoclasts has also been described [19].

Also, further Gla-dependent effects of vitamin K on skeletal biology have been identified. In 2008 a new member of the VDKP group, the upper zone of cartilage and matrix associated protein (UCMA), also termed Gla-rich protein (GRP), was independently identified by three groups. UCMA/GRP is highly expressed in cartilage, in particular in juvenile and resting zone chondrocytes, which secrete the protein to the extracellular matrix [20,21,22]. While zebrafish knockdown experiments indicated a role for UCMA/GRP in skeletal development, Ucma/GRP-deficient mice developed surprisingly normally [23,24]. Under situations challenging the cartilage, in contrast, UCMA/GRP has been found to be protective for cartilage in adult mice. Thus, UCMA/GRP has been shown to protect cartilage from degradation in experimental osteoarthritis and inflammatory arthritis models [25,26].

Further skeleton-associated VDKPs include periostin, protein S, and growth arrest-specific 6 protein (Gas 6). Among other cell types periostin is expressed in osteoblasts and periosteal cells, protein-S is expressed by osteoblasts and GAS6 has been shown to increase osteoclast activity [27,28,29,30,31,32].

This review will focus on the role of vitamin K and in particular of vitamin K-dependent proteins (VKDPs) in bone and cartilage biology.

## 2. Vitamin K

The fat-soluble vitamin K exists in three different forms: Vitamin K1 (Phylloquinone), Vitamin K2 (Menaquinone), and Vitamin K3 (Menadione) [2]. While vitamin K1 and K2 are naturally occurring compounds, vitamin K3 is of artificial origin. Vitamin K1 and K2 are the major forms in the human diet, vitamin K3, in contrast, is only used in animal food due to its comparatively high toxicity (Table 1). In the human body, vitamin K1 is predominantly found in the liver and appears to be mainly involved in the production of coagulation proteins. Vitamin K2, in contrast, is distributed more widely in the body [2].

The three forms of vitamin K share a naphtoquinone group and differ in their side chains. Thus, vitamin K3 lacks a hydrocarbon side chain and is, therefore, water-soluble. In contrast, vitamin K1 and K2 carry hydrocarbon side chains, which render the molecule hydrophobic. The side chain of Vitamin K2 furthermore differs in the number of isoprenyl residues and thus in length. To categorize the different vitamin K2 forms, they are abbreviated as MK-n, with n representing the number of isoprenyl residues [2]. In the human diet, vitamin K1 is mainly found in green vegetables, in particular in kale, spinach, and broccoli. Vitamin K2, in contrast, is mainly found in meat, dairy products, and fermented food, such as the Japanese natto, which is based on fermented soybeans [33].

Vitamin K’s role in blood coagulation is probably the most extensively investigated function of this compound. The key role of vitamin K in coagulation is mediated by its ability to act as a co-factor for an enzyme termed γ-glutamyl carboxylase (GGCX). This enzyme catalyzes the γ-carboxylation of glutamic acid (Glu) residues in vitamin K-dependent proteins (VKDPs) resulting in γ-carboxyglutamic acid residues (Gla). This post-translational modification mediates the Ca2+-binding abilities, which are indispensable for the blood-clotting properties of prothrombin and other coagulation proteins [34,35].

**Figure 1:**
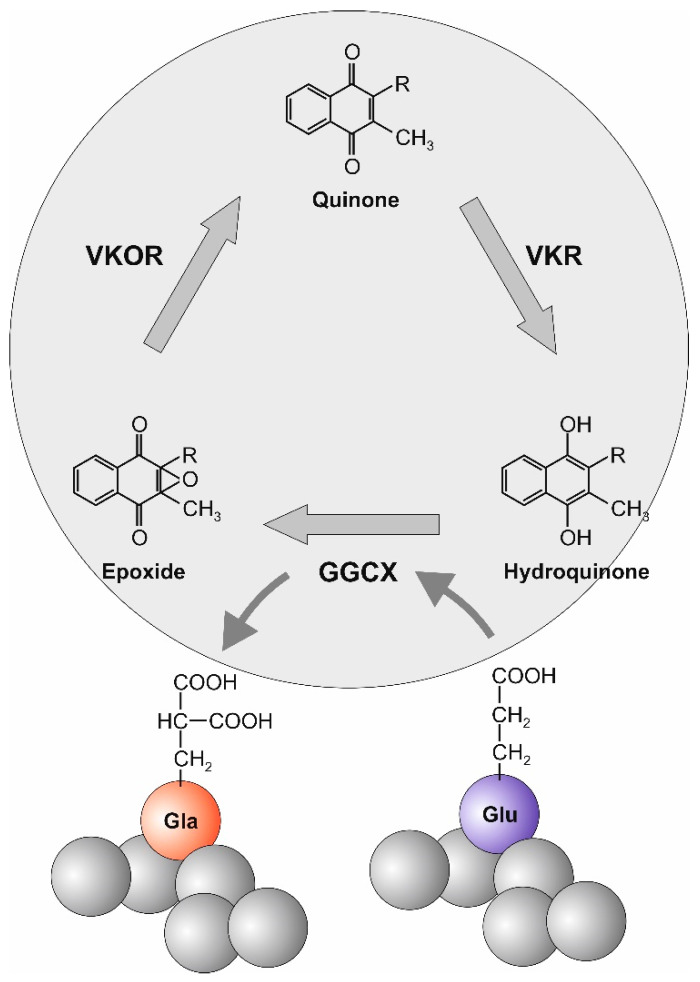
The vitamin K cycle. During γ-carboxylation of glutamyl residues, vitamin K becomes recycled via the vitamin K cycle: (1). The dietary quinone form is reduced to its hydroquinone form by vitamin K reductase (VKR). (2) During γ-carboxylation of Glu residues by γ-glutamyl carboxylase (GGCX) the hydroquinone form of vitamin K is converted to its epoxide form. During this step, a Glu residue is γ-carboxylated to a Gla residue. (3) Vitamin K epoxide reductase converts vitamin K epoxide back to its original form.

During the γ-carboxylation process, vitamin K is not consumed. Instead, the body is able to recycle vitamin K and thereby keep the need for vitamin K uptake relatively low. Vitamin K recycling is accomplished through a process called the vitamin K cycle. Thus, after uptake by the cell, vitamin K is associated with the endoplasmic reticulum where it acts as a co-factor for γ-glutamyl carboxylase (GGCX). Prior to γ-carboxylation of glutamic acid, the dietary quinone form of vitamin K becomes reduced to its hydroquinone form by vitamin K reductase. Thereafter, GGCX-VitK γ-carboxylates glutamic acid residues of Gla-proteins, while vitamin K is converted into its epoxide form. Finally, vitamin K epoxide reductase (VKOR) reduces vitamin K back to its quinone form (Figure 1) [2]. The well-known anticoagulant warfarin suppresses the vitamin K cycle by binding and inhibiting VKOR, thereby leading to depletion of vitamin K quinine and finally blocking the γ-carboxylation of coagulation proteins [36].

## 3. Vitamin K in Skeletal Development and Disease

As described above, the first insights into the involvement of vitamin K in skeletal biology, came from congenital skeletal malformations after treating pregnant patients with the vitamin K antagonist warfarin during their pregnancy [10]. Later vitamin K uptake was shown to be inversely related to bone fracture and osteoporosis risk and vitamin K has been demonstrated to promote bone formation and suppress bone resorption [19,37,38]. Moreover, Vitamin K has also been associated with osteoarthritis and rheumatoid arthritis [39,40,41,42].

Vitamin K-dependent effects on skeletal development and diseases may, however, not be only mediated by VDKPs. Instead, the effects of vitamin K on skeletal biology may be subdivided into VKDP-independent and VDKP-mediated effects.

## 4. VDKP-Independent Effects of Vitamin K on Skeletal Biology

Besides VDKP-dependent effects, bone turnover also appears to be modified by vitamin K mediated but VDKP-independent mechanisms. Thus, vitamin K promotes osteoblast differentiation, while it suppresses osteoclast differentiation by stimulation of osteoprotegerin (OPG) expression and inhibiting the expression of receptor activator of nuclear factor kappa-B ligand (RANKL) [43]. This may be a consequence of vitamin K-dependent but VKDP-independent suppression of NFκB signaling in osteoblasts [19]. Moreover, Igarashi et al. have demonstrated that vitamin K may induce osteoblast differentiation also by pregnane X receptor-mediated transcriptional control of Msx2 [44].

Vitamin K has been shown to ameliorate experimental arthritis in the rat. This effect has been suggested to be mediated by vitamin-K-dependent inhibition of synovial hyperplasia and is considered to be independent of VDKPs [41]. In inflammatory arthritis synovial fibroblast-like cells (SFLCs) exhibit increased proliferation and less apoptosis in response to inflammatory signals. Thus, they form a pannus-like tissue, infiltrating the joint space and secreting further pro-inflammatory cytokines on the one hand, and matrix-degrading enzymes on the other hand [45,46]. Vitamin K has been suggested to enhance apoptosis of FLSCs in inflamed joints [41]. This effect is most likely independent of VKDPs. Thus, vitamin K has been demonstrated to induce reactive oxygen species (ROS) and thereby to increase apoptosis in murine pancreatic acinar cells [47]. Although the direct target of vitamin K in the induction of SFLC apoptosis in inflammatory arthritis has not been established yet, a similar mechanism appears plausible [41]. In inflammatory arthritis, further signaling pathways may be favorably affected by vitamin K. Thus, NFκB signaling, a target of tumor necrosis factor α (TNFα) and other pro-inflammatory cytokines are elevated in arthritis FLSCs and a well-known driver of synovial hyperplasia [46,48]. Interestingly, it had been shown that nuclear factor kappa B (NFκB) signaling in hepatocellular cancer cells is inhibited by vitamin K2, thereby reducing cyclin D1 expression and thus suppressing cell proliferation. A similar mechanism may also contribute to vitamin K-mediated amelioration of experimental arthritis [41].

## 5. Vitamin K-Dependent Proteins in Skeletal Tissues

In skeletal tissues, six different vitamin-dependent proteins are found: Osteocalcin, matrix Gla protein (MGP), Upper Zone of Growth Plate and Cartilage Matrix Associated Protein (Ucma), which is also termed Gla-rich protein (GRP), periostin, protein S, and growth arrest specific 6 protein (GAS6). In bone, four extracellular VKDPs are substantially found. Thus, osteocalcin is specifically expressed by osteoblasts and is considered the most abundant non-collagenous protein in bone [13]. Besides osteocalcin, bone also contains periostin, which is predominantly found in the name-giving periosteum [29]. Protein S and GAS6 are further VKDPs expressed and secreted by osteoblasts to the bone matrix [32,49]. In cartilage, MGP and UCMA/GRP are highly abundant extracellular VKDPs [13,20,21,22].

## 6. Vit K-Dependent Proteins in Skeletal Biology

### 6.1. Osteocalcin (OC; Bone-Gla-Protein)

The thus far best-studied VDKP in bone is osteocalcin. This hydroxyapatite-binding protein is considered the most abundant non-collagenous protein in bone. It is mainly expressed by osteoblasts, but also by odontoblasts and hypertrophic chondrocytes [13,16]. In humans, the transcript is translated to pre-pro-protein of 98 amino acids (11 kDa). The polypeptide contains an N-terminal signal peptide sorting this highly conserved protein for secretion. A pro-peptide harboring a binding site for γ-glutamyl carboxylase is subsequently cleaved releasing the mature osteocalcin protein of 49 amino acids [13,50,51]. In most species, the binding of γ-glutamyl carboxylase to its propeptide induces γ-carboxylation of three Glu residues in osteocalcin. Human osteocalcin, however, remains undercarboxylated at the first of three potential Gla residues [13,51]. Osteocalcin’s Gla residues provide this secreted protein with a high affinity to Ca^2+^ and hydroxyapatite in the mineralized bone matrix [52,53,54]. This raised the hypothesis that osteocalcin may be an important player in bone formation.

Osteocalcin-deficient mice, however, develop surprisingly normally. At birth, they are morphologically indistinguishable from their wild-type littermates. At approximately 6 months after birth, osteocalcin-deficient mice exhibit higher bone mass than wild-type mice. Thus, osteocalcin was identified as a negative regulator of bone formation [55]. Moreover, the biophysical properties of bones in osteocalcin-deficient mice were altered. Thus, hydroxyapatite crystals were less organized, thereby possibly causing increased brittleness [54,56,57]. This indicated that osteocalcin has rather a role in fine-tuning bone synthesis and remodeling than being a pivotal bone constituent for normal skeletal development. In this line, osteocalcin has been identified as a marker for bone formation in post-menopausal osteoporosis [58]. It is noteworthy that the γ-carboxylation status of osteocalcin decreases with age but may be rescued by an increase in vitamin K uptake [59,60]. Elevated serum levels of undercarboxylated osteocalcin have been associated with increased fracture risk in elderly women, indicating that the carboxylation status of osteocalcin is of significance for the bone phenotype [61]. This may be the molecular link of the association of low serum vitamin K levels and decreased bone mineral density and high fracture risk [62,63].

Osteocalcin has also been associated with osteoarthritis (OA). Thus, serum osteocalcin levels were elevated in patients with destructive OA and enhanced expression of osteocalcin in articular cartilage and subchondral bone has been detected in human OA joints [64,65,66]. Interestingly, particularly the serum levels of undercarboxylated osteocalcin were associated with OA, and serum levels of undercarboxylated osteocalcin correlated with serum levels of hyaluronan, which is considered a marker of synovitis [67]. This indicates a link of vitamin K metabolism, osteocalcin and synovial inflammation in osteoarthritis, although the exact mechanism is currently unknown. Interestingly, patients with active rheumatoid arthritis exhibited a decrease in serum osteocalcin levels [68]. Although the physiological significance of alterations in serum osteocalcin levels in OA and RA is currently not known they may reflect the different bone phenotypes in these two joint diseases: serum osteocalcin levels are associated with bone formation, and while OA is rather characterized by an increase of subchondral bone mass (osteophytes, subchondral bone plate thickness), in RA bone resorption and loss of subchondral bone is characteristic [46,69,70].

Although not the focus of this review, it should be mentioned that osteocalcin does not only have direct effects on the biochemical and biophysical properties of the bone matrix but also possesses endocrine functions. Thus, osteocalcin can induce testosterone synthesis in mouse Leydig cells, indicating a role for osteocalcin in male fertility [71]. Moreover, Oury et al. have shown in experiments with osteocalcin-deficient mice that osteocalcin is able to cross the blood-brain barrier where it enhances the synthesis of monoamine neurotransmitters while suppressing GABA synthesis. This prevents anxiety and depression and promotes memory and learning [72]. Most importantly, the endocrine functions of osteocalcin couple bone and glucose metabolism. Thus, in osteocalcin-deficient mice, a decreased β-cell proliferation, glucose intolerance, and insulin resistance has been observed [73]. Moreover, osteocalcin has been shown to induce insulin production in β-cells from pancreatic Langerhans islets and to sensitize adipocytes to insulin by inducing adipocyte expression of adiponectin [73,74]. 

### 6.2. Matrix Gla Protein (MGP)

MGP is a further very well investigated skeletal tissue-associated VDKP. It is mainly expressed in cartilage, but also in fibroblasts and vascular smooth muscle cells [12,13]. Human MGP consists of 84 amino acids (10.6 kDa) and contains a signal peptide sorting the peptide for secretion, and a propeptide including a binding site for γ-glutamyl carboxylase, which is cleaved by a furin-like protease and in its mature for contains 5 Gla residues [13,22,75,76]. This structure provides the protein with a high affinity to Ca2+ and hydroxyapatite [77]. In this line, MGP has been shown to prevent calcification at various sites, including cartilage, vessel walls, skin elastic fibers, and the trabecular meshwork of the human eye. It inhibits vascular calcification [78,79]. Interestingly, the ectopic calcifications observed in MGP-deficient mice resemble those observed in fetal warfarin syndrome [10,11,78].

MGP has been extensively investigated for its role as a protector of vascular and tissue calcification. Thus, mice gene deficient in MGP die at the age of approximately 2 months due to severe vascular calcifications ultimately leading to vessel rupture and massive bleedings [78]. In the vascular system, particularly γ-carboxylated MGP inhibits ectopic mineralization by binding to Ca+ crystals, thereby inhibiting their growth. Moreover, in particular, γ-carboxylated MGP binds to and inhibits BMP-2, a prominent inducer of osteogenesis-like processes leading to calcification [77,80]. This supports the notion that the relationship between ectopic calcifications and low vitamin K serum levels may be linked by MGP. Interestingly, while overexpression of MGP retarded the mineralization of bone in a chick system, excessive elevation of MGP serum levels by overexpression could not reduce vascular calcification in mice, indicating that during physiological conditions MGP levels are already optimal for controlling vascular calcification [14,79].

In the joint compartment, MGP plays a significant role in the progression of osteoarthritis (OA). Thus, low serum levels of vitamin K are associated with OA and OA chondrocytes secrete mainly uncarboxylated MGP [39,81]. Moreover, MGP-dependent inhibition of BMP-2 and calcification, and polymorphisms in the MGP gene associated with OA indicate a role for MGP in OA [80,82,83]. The carboxylation status of MGP in different forms of arthritis is currently under debate. Thus, Silaghi et al. observed that uncarboxylated MGP levels in synovial fluid were higher in inflammatory arthritis than in OA. In the serum it was vice versa: uncarboxylated MGP serum levels are higher in OA patients than in inflammatory arthritis patients [84]. In contrast, Bing and Feng observed serum uncarboxylated MGP to be reduced in OA patients and synovial fluid uncarboxylated MGP levels to be negatively correlated with OA disease severity [85]. Uncarboxylated MGP levels are furthermore associated with higher fracture risk and osteoporosis [86]. On the other hand, overexpression of MGP in mice (which probably results in high levels of γ-carboxylated MGP) ameliorated ovarectomy-induced osteoporosis [87]. Together these works demonstrate a putative role for MGP in ectopic mineralization and a particular significance of vitamin K-dependent γ-carboxylation of this protein as well in soft tissue as in skeletal homeostasis.

### 6.3. UCMA/GRP

In 2018 the latest member of VDKPs, upper zone of cartilage and matrix associated protein (UCMA)/Gla-Rich Protein (GRP) was independently discovered and first described by three groups at virtually the same time: our group (Surmann-Schmitt et al.) identified UCMA/GRP in murine chondrocytes, Tagariello et al. in human growth plate cartilage and Viegas et al. in sturgeon cartilage [20,21,22].

In mice, our group and Tagariello et al. found a highly specific gene expression of UCMA/GRP in juvenile and articular cartilage, detected by RNA in situ hybridization, while Viego et al. observed a gene expression pattern somewhat less confined to chondrocytes in sturgeon and rat: in these species UCMA/GRP gene expression was also detected in hypertrophic chondrocytes and osteoblasts [20,21,22]. The question of cartilage-specificity is currently still a matter of debate. Thus, Lee et al. described UCMA/GRP gene expression also in the murine osteogenic cell line MC3T3-E1 and in primary calvarial osteoblasts detected by real-time RT-PCR [88]. In fact, our group had also detected UCMA/GRP in MC3T3-E1 by RT-PCR. However, UCMA/GRP was detected in MC3T3-E1 cells at negligible levels compared to chondrogenic MC615 cells or primary chondrocytes [20]. In fact, UCMA/GRP transcripts detected by Lee et al. in MC3T3-E1 cells and primary murine calvarial osteoblasts were at a similarly low level as collagen2a1, a gene considered highly specific for cartilage [88]. Moreover, a UCMA/GRP reporter mouse, harboring a lacZ cassette integrated into UCMA/GRP exon 1, did not indicate gene expression in any other tissue than cartilage, in particular not in bone, and comparing UCMA mRNA levels in epiphysial chondrocytes, calvarial cells, and cortical bone from mice aged 3 to 18 days using real-time RT PCR revealed negligible Ucma gene expression in the bone samples as compared to cartilage samples [24,26].

Ucma/GRP is highly conserved in vertebrates—except birds—and it is transcribed into a set of different splice variants [20,21,22,89,90]. Currently, however, it is not clear, which physiological significance the different splice variants possess. The full-length open reading frame in mouse codes for 138 amino acids with a predicted molecular mass of 16.5 kDa. The translated polypeptide contains a signal peptide for secretion and a cleavage site for furin-like proteases [20,21,22]. During or after secretion the amino-terminal pro-peptide, which contains a binding site for γ-glutamyl carboxylase, is cleaved off and the C-terminal mature polypeptide is released to the extracellular matrix [20,21,22]. Usually, Ucma/GRP is γ-carboxylated at all 14 Glu residues of its amino acid chain, providing the protein with a high affinity to Ca^2+^ [22]. Furthermore, Ucma/GRP is sulfated at up to two tyrosine residues, which is typical for extracellular matrix proteins, such as VDKPs or leucine-rich repeat proteoglycans, and is considered to promote protein-protein interactions [20,91,92]. In fact, we could later demonstrate that Ucma/GRP is binding to collagen type II with high affinity [26].

These highly interesting features raised our interest in the physiological function of Ucma/GRP and pointed to the role of Ucma in skeletal development. Indeed, knockdown of Ucma in zebrafish resulted in severe skeletal malformations and decreased collagen type II and aggrecan content in cartilage, supporting the concept of an important function in skeletal development. Moreover, these malformations were similar to those observed in zebrafish in which γ-carboxylation of Glu residues had been blocked by warfarin, indicating that warfarin-induced developmental effects may be mediated by partial or complete lack of Ucma glutamyl γ-carboxylation [23]. Together these findings encouraged us to generate and investigate a gene-targeted mouse strain deficient in Ucma/GRP. Surprisingly, however, these Ucma/GRP-deficient mice developed normally without any impairment of skeletal development [24]. This indicated that Ucma is not required for normal embryonic and post-natal development in mice, and maybe also not in other mammals. On the other hand, Michou et al. identified Ucma/GCP SNPs to be correlated with Paget’s disease during a genetic association study [93]. This indicated an in vivo role for Ucma in bone-associated diseases in humans and in particular supported the concept of Ucma acting as a modifier of calcification processes. Moreover, effects of Ucma/GRP have also been demonstrated on the cellular level: thus during our initial investigation, we observed recombinant Ucma/GRP to inhibit osteoblastic differentiation [20]. Interestingly, Lee et al. observed the opposite for Ucma/GRP overexpressed in osteoblasts: Ucma/GRP overexpression in MC3T3-E2 cells promoted osteogenic differentiation [88]. These differences may be explained by different post-translational modifications of Ucma/GRP and may, in particular, reflect a difference in γ-carboxylation. These findings raise of cause the question of the origin of Ucma/GRP that my act on bone in the body. Our analyses indicated only marginal levels of Ucma/GRP in bone [20,24,26]. Nevertheless, we also found Ucma/GRP to be secreted to the extracellular space, where it readily migrates through the matrix [20,21,24,26]. Thereby, cartilage-born Ucma/GRP may act also on tissues other than cartilage.

Ucma/GRP is also expressed in calcifying vascular smooth muscle cells, which undergo a differentiation similar to osteogenesis. Moreover, Ucma/GRP is present in calcified atherosclerotic plaques. Interestingly, mice deficient in Ucma/GRP exhibited increased levels of vascular calcification, indicating that Ucma/GRP may act as a suppressor of vascular calcification [94]. Moreover, Ucma/GRP has been detected in serum and in circulating calciprotein particles (CCPs), which play an important role in calcification processes, particularly in vascular calcification [95,96]. Chronic kidney disease (CKD) patients often suffer from enhanced vascular calcification. Notably, CCPs from patients with severe CKD have been shown to contain less GRP. These findings further support the concept of Ucma/GRP as a calcification inhibitor [95]. Recently, low serum Ucma/GRP levels have been shown as a marker of vascular calcification in CKD patients [97].

Looking back to the skeleton, we and others investigated a potential role for Ucma in typical cartilage-associated diseases. Thus, Cavaco et al. found that carboxylated Ucma/GRP inhibited calcification of the extracellular matrix in a synoviocyte/chondrocyte cell system under osteoarthritis (OA)-like conditions [98]. Our group analyzed the progression of OA in the DMM (destabilization of the medial meniscus) mouse model for OA. In fact, we observed a profound exacerbation of OA in Ucma/GRP-deficient mice, pointing to a protective effect of Ucma/GRP on cartilage [26]. Searching for the molecular mechanism of this protective effect we found three mechanisms that may contribute to cartilage protection: (i) Ucma/GRP is binding to the cartilage-associated collagen types II, IX, and XI with high affinity. This interaction may support the interconnection of the cartilage matrix and promote its mechanical rigidity. (ii) In articular cartilage of Ucma-deficient mice with experimental OA we observed a significant increase in chondrocyte apoptosis or cell death. (iii) The potentially most important feature of Ucma in terms of cartilage protection is its ability to bind to and inhibit aggrecanases of the ADAMTS family, namely ADAMTS4 and -5. In line with this finding, Ucma-deficient mice with experimental OA exhibited elevated levels of ADAMTS-derived aggrecan cleavage products [25,26]. Our studies were recently supported by a work from Okuyan et al. who demonstrated that intraarticular injections of Ucma/GRP in the knee joints of rats with experimental OA ameliorated cartilage degeneration [99]. Interestingly, also, Cavaco et al. have shown that Ucma/GRP downregulated the expression of pro-inflammatory cytokines and that only γ-carboxylated Ucma/GRP inhibited the calcification of the extracellular matrix in vitro. Moreover, they observed that chondrocytes induce Ucma/GRP expression under OA conditions including treatment with IL-1β [98]. This is in line with our observations during DMM-induced OA in mice, showing an increase in Ucma/GRP expressing articular chondrocytes [26]. Ucma/GRP levels in synovial fluid have also been shown to be elevated in OA patients and they positively correlated to disease stage [100]. Together these findings suggest that Ucma/GRP is a potent protector of cartilage during OA and the upregulation of Ucma/GRP in articular cartilage during OA may represent a repair response to damaged cartilage. These findings may also indicate that Ucma/GRP might at least contribute to the association of vitamin K with osteoarthritis [39,40]

Interestingly, the effect of Ucma/GRP in experimental OA was not limited to the cartilage compartment. Thus, we observed reduced osteophyte formation and subchondral bone plate thickness in Ucma/GRP-deficient mice with experimental OA. This was accompanied not only by reduced osteoblast counts but also reduced osteoclast numbers in Ucma/GRP-deficient mice with experimental OA [26]. As mentioned above, Ucma/GRP has the potential to modify osteoblast differentiation in vitro [20,88]. The findings in experimental OA support this notion, demonstrating that in vivo Ucma/GRP appears to promote osteoblast formation. Osteoclasts were also affected by Ucma/GRP-deficiency under OA conditions. In fact, we showed that recombinant Ucma, as well as wild-type cartilage explants, promoted in vitro osteoclastogenesis. Cartilage explants from Ucma/GRP-deficient mice, however, exhibited a strikingly reduced potential to promote in vitro osteoclastogenesis. The nature of this pro-osteoclastogenesis mechanism is currently not clear, yet. The effect may, however, be mediated by p38 and ERK, since recombinant Ucma/GRP induced the phosphorylation of these MAP kinases in osteoclast precursors [20,88].

In inflammatory arthritis, Ucma/GRP appears to play a similar role as a protector of cartilage and a promoter of bone turnover. In a mouse model for inflammatory art, hritis we observed strikingly exacerbated cartilage destruction and reduced osteophyte formation in Ucma/GRP-deficient mice. The cartilage-protective effect was also supported by the finding that systemic administration of recombinant Ucma/GRP substantially ameliorated cartilage damage. In contrast, joint inflammation was not affected by Ucma/GRP [25]. Thus, like in OA, Ucma may also provide a link for the ameliorating effects of vitamin K in arthritis [41,42].

In total, these findings present Ucma/GRP as a multifunctional secreted VDKP that controls ectopic calcification, promotes bone turnover, and protects cartilage under pathologic conditions such as chronic kidney disease, osteoarthritis, and inflammatory arthritis. 

### 6.4. Periostin, Growth Arrest-Specific Protein 6 (Gas6), and Protein S

The role of these VDKPs in skeletal biology has not been studied as extensively. Periostin is expressed in osteoblasts and periosteal cells among other cell types and is related to skeletal development, bone fracture healing, osteoporosis, and osteoarthritis [28,29,30,31,101]_ENREF_30. Periostin-deficient mice exhibit a disorganized epiphyseal growth plate architecture resulting in growth retardation, particularly characterized by shorter long bones [29,102]. In part, this may be explained by the finding that periostin binds to collagen I, and periostin-deficient mice exhibit aberrant collagen I fibril formation [103]. Moreover, periostin-deficient mice exhibit increased sclerostin expression. Sclerostin is a potent inhibitor of bone growth in response to mechanical load. Therefore, elevated sclerostin levels in periostin-deficient mice may contribute to a loss of bone mass in these animals [102]. Periostin has also been associated with OA. Thus, serum and synovial fluid levels of periostin have been reported to be increased in OA [30,104,105]. Interestingly, Attur et al. have observed experimental OA to be ameliorated in periostin-deficient mice [106]. These findings may introduce periostin as a promising novel target for future disease-modifying therapies.

Protein-S is also expressed by osteoblasts and secreted to the bone matrix [32]. Shortage in protein-S has been associated with osteopenia [107]. In contrast, GAS6 has been shown to increase osteoclast activity thereby promoting bone resorption [27].

## 7. Prospects

Several lines of evidence demonstrate the importance of Vitamin K for skeletal development and health. Vitamin K is not particularly toxic and a high intake of vitamin K is generally considered beneficial. Thus, high vitamin K intake is negatively associated with osteoporosis while vitamin K antagonists have been shown to increase the risk for osteoporosis and osteoarthritis [37,38,39,40]. Moreover, vitamin K possesses some anti-tumorigenic properties [5,8]. This may suggest a benefit of dietary supplementation with vitamin K. In fact, vitamin K is approved for the treatment of osteoporosis in Japan [2]. However, a recent study demonstrated a correlation of high vitamin K intake with elevated risk for breast cancer [9]. This indicates that dietary vitamin K supplementation needs to be further evaluated in order to obtain a comprehensive understanding of associated risks and benefits.

**Figure 2:**
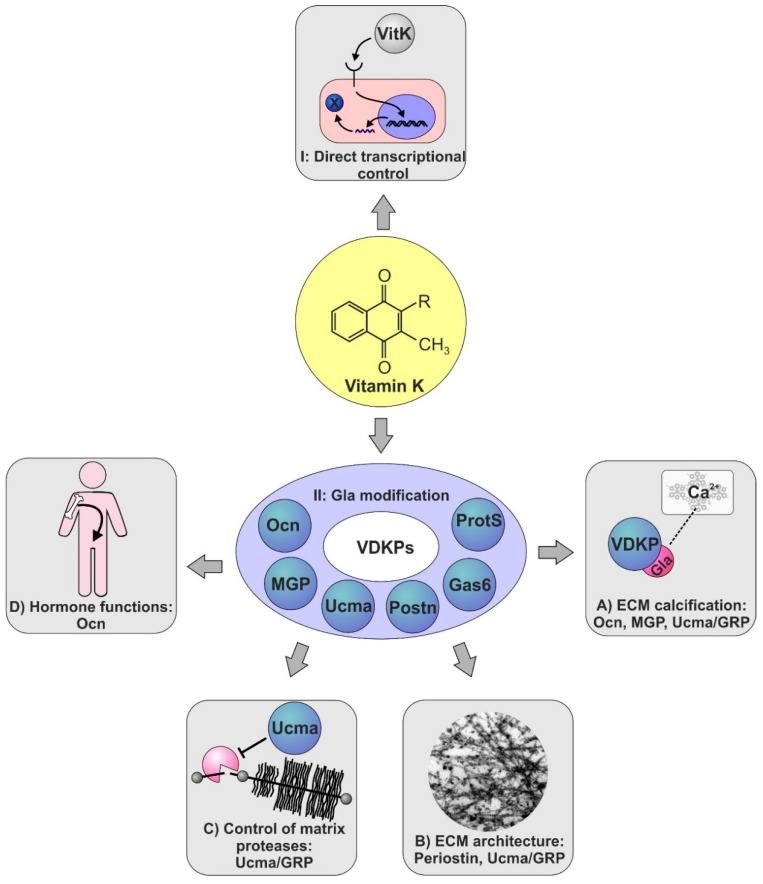
Vitamin K-dependent pathways in skeletal biology. Vitamin K applies multiple molecular pathways in order to affect skeletal biology: I.: Vitamin K may affect cartilage and bone by binding to cell surface receptors, such as the pregnane X receptor. This results in the modulation of downstream signaling pathways ultimately modifying gene expression (e.g., of Msx2). II.: Vitamin K may exert actions on skeletal tissues by acting as a co-factor in the γ-carboxylation of VKDPs. VDKPs, in turn, apply further pathways to affect cartilage and bone biology: (**A**) Through their Gla-residues VKDPs can bind to calcium crystals and thereby modulate calcification processes in bone and soft tissues. This has been particularly investigated for osteocalcin, MGP, and Ucma. (**B**) The second mode of action of VKDPs is the interaction with extracellular matrix proteins, supporting the organization of the ultrastructural matrix architecture (shown, e.g., for periostin and suspected for Ucma/GRP). (**C**) Inhibition of matrix proteases has been shown for Ucma/Grp and mediates a protective effect of Ucma/GRP against cartilage matrix degradation. (**D**) Finally, hormone-like effects of osteocalcin have been shown to mediate crosstalk between bone and glucose metabolism. ECM: extracellular matrix; Postn: periostin; ProtS: protein S; VitK: Vitamin K; X: downstream target gene/protein of Vitamin K.

Vitamin K may exert its effect on skeletal tissues either directly, by affecting skeletal gene expression or as a co-factor in the post-translational modification of VKDPs. VDKPs are a group of proteins, which play important roles in skeletal development (such as MGP) physiological homeostasis of the skeleton (such as osteocalcin, MGP, and periostin), or under pathological conditions of the skeletal system (such as osteocalcin, MGP, Ucma/GRP periostin). Their roles are often, but not solely mediated by calcium-binding through the VKDP’s Gla residues and the control of calcification processes (refer to Section 4, Section 5 and Section 6, Figure 2). 

Particularly, Osteocalcin and MGP, but also Ucma/GRP are involved in calcification processes in bone and other tissues. Thereby, they contribute to the physiological homeostasis of these tissues. Of particular focus is currently the ability of both MGP and Ucma/GRP, to ameliorate blood vessel calcification [86,94]. However, MGP and Ucma/GRP may also be involved in the control of calcification processes in skeletal diseases. Thus, these proteins play roles in osteoporosis and/or osteoarthritis [81,86,93,98]. These findings open a new field for targeting calcification-dependent diseases. However, while most of these findings have been obtained using mouse and rat models, further studies will have to evaluate, whether these mechanisms may be translated to the situation in humans.

Beyond their roles in calcification processes, VKDPs often apply further modes of action. Thus, osteocalcin serves as a hormone in the cross-talk between bone and bone metabolism as well and affects male fertility [71,73]. Ucma/GRP may develop promising therapeutical potential in the control of cartilage degeneration during degenerative and inflammatory joint diseases by inhibition of aggrecanases [25,26]. Thus, aggrecanases ADAMTS-4 and ADAMTS-5 have been shown to mediate pivotal steps in cartilage degeneration during both animal models and human degenerative and inflammatory joint diseases [108,109,110,111]. Consequently, aggrecanase inhibitors are being extensively investigated for their therapeutical use against cartilage degeneration [111,112]. However, important roles for ADAMTS-4 and -5 in the homeostasis of tissues other than cartilage may limit the therapeutical options for aggrecanases inhibitors. Thus, it had been shown that ADAMTS-5 ameliorates atherosclerosis-related deposition of extracellular matrix proteins to the vessel wall [113]. In that respect, Ucma/GRP may provide the advantage that its ability to bind to cartilage-specific collagen type II might target systemically administered Ucma/GRP to the cartilage compartment [26].

Further studies will have to evaluate, whether the cartilage-protective effect of Ucma/GRP may be translated to the human situation. Finally, interactions with other proteins of the extracellular matrix may contribute to VKDP-dependent mechanisms that determine skeletal homeostasis. Thus, Ucma/GRP has been shown to bind to cartilage-specific collagen type II and periostin physically interacts with collagen type I, abundantly present in bone, tendon, and heart valves [26,103]. In mice, lack of periostin has been shown to result in aberrant collagen fibril formation, indicating a role for periostin in the ultrastructural architecture of the extracellular matrix in bone and other collagen I-rich tissues [103]. It remains to be elucidated, whether Ucma/GRP has a similar role in the architecture of the cartilage matrix.

Together, VKDPs display a wide variety of pivotal functions in development, homeostasis, and disease of cartilage and bone. Thereby, they represent promising targets for the development of specific disease-modifying drugs. Nevertheless, the concepts summarized here, have predominantly been developed in animal models and their potential translation to the human system needs to be carefully investigated.

## Figures and Tables

**Table 1:** Three forms of vitamin K.

	Vitamin K1 (Phylloquinone)	Vitamin K2 (Menaquinone)	Vitamin K3 (Menadione)
Synonym		MK-n	
Molecular Structure	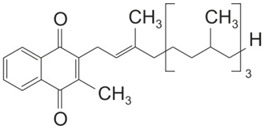	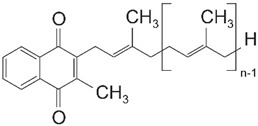	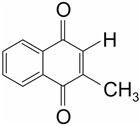
Origin	Vegetables	Fermented foods	synthetic
Usage	Human diet	Human diet	Widely used in animal food but not for human diet

## Data Availability

Not applicable.
